# Socioeconomic health inequality in malaria indicators in rural western Kenya: evidence from a household malaria survey on burden and care-seeking behaviour

**DOI:** 10.1186/s12936-018-2319-0

**Published:** 2018-04-16

**Authors:** Vincent Were, Ann M. Buff, Meghna Desai, Simon Kariuki, Aaron Samuels, Feiko O. ter Kuile, Penelope A. Phillips-Howard, S. Patrick Kachur, Louis Niessen

**Affiliations:** 1 Centre for Global Health Research, Kenya Medical Research Institute, Kisumu, Kenya; 2 Malaria Branch, Division of Parasitic Diseases and Malaria,Center for Global Health, Centers for Disease Control and Prevention, Atlanta, USA; 3 U.S. President’s Malaria Initiative, Nairobi, Kenya; 4 Liverpool School of Tropical Medicine, Liverpool, UK

**Keywords:** Socioeconomic, Malaria, Medication, Inequalities, Kenya

## Abstract

**Background:**

Health inequality is a recognized barrier to achieving health-related development goals. Health-equality data are essential for evidence-based planning and assessing the effectiveness of initiatives to promote equity. Such data have been captured but have not always been analysed or used to manage programming. Health data were examined for microeconomic differences in malaria indices and associated malaria control initiatives in western Kenya.

**Methods:**

Data was analysed from a malaria cross-sectional survey conducted in July 2012 among 2719 people in 1063 households in Siaya County, Kenya. Demographic factors, history of fever, malaria parasitaemia, malaria medication usage, insecticide-treated net (ITN) use and expenditure on malaria medications were collected. A composite socioeconomic status score was created using multiple correspondence analyses (MCA) of household assets; households were classified into wealth quintiles and dichotomized into poorest (lowest 3 quintiles; 60%) or less-poor (highest 2 quintiles; 40%). Prevalence rates were calculated using generalized linear modelling.

**Results:**

Overall prevalence of malaria infection was 34.1%, with significantly higher prevalence in the poorest compared to less-poor households (37.5% versus 29.2%, adjusted prevalence ratio [aPR] 1.23; 95% CI = 1.08–1.41, p = 0.002). Care seeking (aPR = 0.95; 95% CI 0.87–1.04, p = 0.229), medication use (aPR = 0.94; 95% CI 0.87–1.00, p = 0.087) and ITN use (aPR = 0.96; 95% CI = 0.87–1.05, p = 0.397) were similar between households. Among all persons surveyed, 36.4% reported taking malaria medicines in the prior 2 weeks; 92% took artemether-lumefantrine, the recommended first-line malaria medication. In the poorest households, 4.9% used non-recommended medicines compared to 3.5% in less-poor (p = 0.332). Mean and standard deviation [SD] for expenditure on all malaria medications per person was US$0.38 [US$0.50]; the mean was US$0.35 [US$0.52] amongst the poorest households and US$0.40 [US$0.55] in less-poor households (p = 0.076). Expenditure on non-recommended malaria medicine was significantly higher in the poorest (mean US$1.36 [US$0.91]) compared to less-poor households (mean US$0.98 [US$0.80]; p = 0.039).

**Conclusions:**

Inequalities in malaria infection and expenditures on potentially ineffective malaria medication between the poorest and less-poor households were evident in rural western Kenya. Findings highlight the benefits of using MCA to assess and monitor the health-equity impact of malaria prevention and control efforts at the microeconomic level.

## Background

Malaria is one of the most important diseases in many low- and middle-income countries, primarily affecting children and pregnant women in sub-Saharan Africa. In 1999, approximately 60% of global malaria deaths were concentrated among the poorest 20% of the global population [1]. In sub-Saharan Africa, 3.1% of all disability-adjusted life-years (DALYs) were lost to malaria in 2002 [2]. Although preventable and treatable, the number of deaths due to malaria remains high. In 2015, there were an estimated 429,000 malaria deaths (range 235,000–639,000) worldwide, and most (92%) of these deaths occurred in the Africa [3]. In Kenya, despite remarkable achievements in malaria prevention and control over the last 10 years, malaria remains a leading cause of morbidity and mortality [4]. In 2015, while the prevalence of microscopically-confirmed malaria was 8% amongst children less than 15 years (13% by malaria rapid diagnostic test [RDT]) nationally, it was 27% (43% by malaria RDT) in the lake-endemic region of western Kenya [5].

The relationship between malaria disease and poverty has often been described as a vicious cycle and whether malaria infection is a consequence of or a cause for low household socioeconomic status (SES) has been debated for decades [6]. In a systematic review of nine studies to establish the relationship between malaria and poverty, two studies found a significant relationship between poverty and malaria, four studies found no significant relationship and three studies demonstrated mixed results [7]. Malaria also imposes substantial costs to individuals, households and governments. Globally in 2015, total funding for malaria control and elimination efforts was estimated at US$2.9 billion; governments in malaria-endemic countries provided 32% of the total funding, of which 65% or US$612 million was expenditure by national malaria control programmes for programme implementation and 35% or US$332 million was expenditure on health service delivery [3]. In high malaria-transmission settings in Kenya where the average annual household expenditure was less than US$800 [8], households spent an average of US$10 (range US$9–12) monthly and approximately US$120 (range US$108–144) annually on malaria treatment in 2010 [9].

The microeconomic relationship between malaria burden and composite wealth indices is also mixed and contradictory [7]. A study among Tanzanian children, using principal component analysis (PCA) to rank households, established that malaria was associated with household SES when SES was the dependent variable, but an individual’s SES was not associated with malaria risk when malaria was the dependent variable [6]. Another study in Tanzania, which investigated causality between malaria and SES, established that the higher the household wealth quintile the lower the prevalence of malaria in individuals in the household [10]. The lack of consistent findings may partly reflect the inherent difficulty in measuring SES and differences in the populations studied, methods used to measure malaria infection and malaria intensity in the study areas [7, 11–13].

Various methodologies for assessing SES employing broad quantitative and qualitative aspects of poverty have been recommended including PCA, polychoric PCA and multiple component analysis (MCA) [14–19]. A study from rural western Kenya compared the three methods of ranking households into SES quintiles and established that although the methods produced similar results, MCA gave the highest percentage of the total variation for the household asset variables and thus the largest weights for the variables. The study concluded that MCA was a better model for generating asset weights than PCA or polychoric PCA [20]. The study further conducted comparison between ordinary PCA and MCA and established that 93% of households were placed in the same quintile by both methods, 87% of the households by ordinary PCA and Polychoric, and 91% by Polychoric and MCA and that ordinary PCA asset index was statistically significantly correlated with the index based on MCA (r = 0.997, p < 0.01) [20]. The MCA model also allows both quantitative and qualitative variables, which is not possible with traditional PCA methods [20–22]. However, studies using MCA methods to investigate socioeconomic inequalities related to malaria indicators are lacking.

Health inequality data, including malaria parasitaemia prevalence, use of malaria prevention and treatment interventions and expenditures on malaria medication, are often collected but not analysed from an economic or equity perspective. Yet, such data and analysis are important for monitoring health inequalities and the impact of malaria control interventions at the microeconomic level. The aim of this study was to establish the relationship between household SES and inequalities in malaria-related health indicators including morbidity, use of insecticide-treated nets (ITNs), care seeking, and expenditure on malaria medications in a malaria-endemic area of rural western Kenya.

## Methods

### Study site

A community-based cross-sectional survey was conducted in mid-2012, a year after a mass ITN distribution in Siaya County. The survey was conducted within the Kenya Medical Research Institute (KEMRI) and Centers for Disease Control and Prevention (CDC) health and demographic surveillance system (HDSS) in Siaya County, western Kenya. The HDSS has been described in detail elsewhere [20, 23–25]. Briefly, the HDSS area covers a population of approximately 223,000 people residing in 393 villages in three sub-counties of Siaya County, spread over approximately 700 km^2^ along the shores of Lake Victoria. The vast majority of the population earn their living through subsistence farming and fishing. Residents of the HDSS were visited in their homes every 4 months to record births, deaths, pregnancies, immigration and out-migration. Health indicators in Siaya County, formerly part of Nyanza Province, are poor compared to the national averages [26, 27]. Nyanza Province had the highest rate of child mortality at 72 deaths per 1000 live births in 2008–2009, and an estimated 60% of the population lived below poverty line during the study period [26, 27].

### Population, sampling strategy and sample size

The sampling frame obtained from the HDSS included all households with children < 5 years of age because many malaria interventions target this age group. Households were selected for participation by systematic random sampling, stratified by sub-county (i.e., Rarieda, Gem or Siaya). The sampling interval was calculated by dividing the total number of selected compounds by the target sample size. A random start number was selected from the ordered compound listing. Households were selected based on systematically adding the sampling interval to the random start number until the required sample size had been achieved. In total, 998 compounds comprising 1063 households were sampled. A sub-sample of all household members were surveyed (5–14 years and 15 + years), except for children < 5 years, who for ethical reasons were all included. If an individual of any age group (< 5 years, 5–14 years and ≥ 15 years) was sampled in a household, then all children < 5 in that household were also included in the survey.

### Data collection

Study participants were interviewed face-to-face by trained field staff using a structured questionnaire, programmed into a personal digital assistant, to collect data on demographic factors, socioeconomic factors including asset ownership and utilities, ITN ownership and usage night before the survey, history of fever in the past 14 days, care-seeking behaviours and medication use in the past 14 days. A finger prick blood specimen was obtained from all individuals in the sampled households; haemoglobin was measured by HemoCue^®^ (Ängelholm, Sweden) and the presence of malaria parasitaemia was evaluated using RDT (Carestart™ Malaria HRP-2/pLDH (Pf/PAN) Combo, Somerset, NJ, USA). Individuals with a positive malaria RDT were treated in accordance with the 2010 Kenya national malaria treatment guidelines for uncomplicated malaria while complicated cases were referred to nearby health facilities for treatment [28]. Additionally, thick and thin blood smears were taken for screening, malaria species’ identification and enumeration of parasite density.

Medication prices used to estimate expenditures were obtained from a separate survey in the same area, which assessed the availability and cost of antimalarial medications in September 2013 [29]. Medication prices were estimated using the local prices in Kenya shillings and converted to US dollars using the October 2013 exchange rate of 85 Kenya shillings to US$1.00 [29]. Non-recommended medications for uncomplicated malaria included amodiaquine, chloroquine, sulfadoxine-pyrimethamine, and quinine (not recommended for use by non-pregnant women). In Kenya, quinine is only recommended as a first-line malaria medication for women in the first trimester of pregnancy [28]. The variables used to generate household SES index included the occupation of household head (which included; doing business, commercial, farming, housewife, salaried worker, skilled labour, unskilled labour and subsistence farming) primary source of drinking water, type of cooking fuel, ownership of household assets (e.g., lantern lamp, radio, television, bicycle) and ownership of livestock (e.g., cattle, chicken, pigs, donkey) [20].

### Data management and analysis

Data were downloaded into a Microsoft Access (Version 2010, Microsoft, and Seattle, WA, USA) database for management. Laboratory analyses of microscopy results were recorded in an Excel (Version 2010, Microsoft, Seattle, WA, USA) spreadsheet. All the datasets underwent validation and consistency checks to identify and resolve errors before they were merged using the HDSS unique identifiers or sample codes as appropriate.

Using the MCA model, households were characterized into five socio-economic quintiles with the first quintile as the poorest and the fifth quintile as the least-poor based on household assets, utilities and occupation [20, 22]. A generalized linear model, customized with a log-link function, was used to estimate and compare adjusted prevalence ratios (aPR). Dependent variables included malaria infection, care seeking, medication and ITN use, while SES, study areas (i.e., sub-counties), sex and age groups (< 5, 5–14 and ≥ 15 years) were included as independent variables. As described in detail elsewhere, SES quintiles were aggregated into dichotomous groups [9]. A binary variable was created with the first three SES quintiles (i.e., poorest, second and third poorest) grouped as the ‘poorest’ category and the fourth and fifth quintiles grouped into the ‘less-poor’ category, with the latter as the reference category in the models. Proportions and 95% confidence intervals were generated, and p values < 0.05 were considered statistically significant. Proportions were compared using Fisher’s exact test, and the price of medications were compared using a generalized linear model. Medians and interquartile ranges were generated if data were not normally distributed; medication expenditures were compared using Wilcoxon rank-sum test because price data were not normally distributed.

### Ethics, consent and permissions

The HDSS protocol and consent procedures, including surveillance, were approved by KEMRI (SSC#1801) and CDC (#3308) institutional review boards (IRB) annually. The malaria-specific survey, including collection of blood samples, received approval from the KEMRI scientific steering committee (#2031) and CDC IRB (#6012). Written consent was obtained in the local language prior to administration of the questionnaires.

## Results

### Characteristics of study participants

A total of 1063 households and 2719 individuals were surveyed in July 2012, approximately 1 year after a mass ITN distribution in the study area. Participants ages were categorized into < 5 years (56.8%; n = 1545), 5–14 (17.4%; n = 437), and ≥ 15 years (25.8%; n = 701) (Table 1).

**Table 1:** Sociodemographic characteristics of study population in Siaya County, Kenya, 2012

Categories	n^a^	Percent
Age groups (years)		
< 5	1545	56.8
5–14	437	17.4
≥ 15	701	25.8
Sex		
Female	1494	54.9
Male	1225	45.1
Wealth quintiles (SES)		
1 (Poorest)	458	20.6
2	434	19.5
3	459	20.6
4	436	19.6
5 (Least poor)	441	19.8
Sub-counties		
Rarieda	834	30.7
Gem	872	32.0
Siaya	1013	37.3

^**a**^n = 2719 total population surveyed

### Descriptive epidemiology

The prevalence of malaria parasitaemia by microscopy was 34.1% overall, 34.4% among children < 5 years, 54.8% in 5–14 year olds and 19.3% in persons aged ≥ 15 years (Table 2). Fever in the 14 days prior to the survey was self-reported by 53.9% (n = 1463) of the survey population; this was highest (60.6%) among children aged < 5 years (Table 2). Of those reporting fever, 70.4% (n = 1032) had sought care. Of those who sought care, 51.8% sought care from health facilities, 30.4% from pharmacies, and 15.4% from shops. Use of any medication among those who reported having fever was 77.3% overall, with the highest proportion among young children (81.9%), and lowest (65.2%) among persons aged ≥ 15 years. Overall, 64.9% of the population reported ITN use the night prior to the survey (Table 2).

**Table 2:** Descriptive epidemiology of malaria-related indicators in Siaya County, Kenya, 2012

	< 5 years n (%)	5–14 years n (%)	≥ 15 years n (%)	Total n (%)
Fever in prior 2 weeks^a^	935 (60.6)	209 (44.2)	319 (45.5)	1463 (53.9)
Malaria parasitaemia	532 (34.4)	259 (54.8)	135 (19.3)	926 (34.1)
Care seeking	708 (75.6)	134 (64.1)	190 (59.6)	1032 (70.4)
Medication usage^b^	766 (81.9)	157 (75.1)	208 (65.2)	1131 (77.3)
ITN use	1033 (66.9)	251 (53.1)	481 (68.6)	1765 (64.9)

*ITN* insecticide-treated net

^**a**^Self-reported fever

^**b**^Of 1463 persons who reported having fever, 1131 took medication

### Multivariable analysis

Overall, 37.6% of persons from the poorest households had malaria infection compared to 29.2% of persons from less-poor households (adjusted prevalence ratio [aPR] = 1.23; 95% CI = 1.08–1.41, p = 0.002), when adjusting for age, geographic area, gender and ITN use (Table 3). In multivariate analysis of care seeking (n = 1182), children < 5 years were significantly more likely to seek care (aPR = 1.27; 95% CI = 1.14–1.41, p < 0.001) compared to adults ≥ 15 years. There were no significant differences in care seeking by SES, gender or geographic area (Table 4). Among persons who reported fever in the prior 14 days, prevalence of medication use was significantly higher in children < 5 years (aPR = 1.27; 95% CI = 1.15–1.40, p < 0.001) compared to adults ≥ 15 years. The poorest persons reported less medication use, but it was not significantly different compared to the less-poor (aPR 0.94, 95% CI = 0.88–1.0; p = 0.05) (Table 4).

**Table 3:** Multivariate analysis of socioeconomic status and association with malaria infection in Siaya, Kenya, 2012

Characteristic	n	N	Malaria parasitaemia percent	Adjusted prevalence ratio	95% confidence interval		*p* value
Overall	926	2228	34.1				
SES							
Poorest^a^	401	1070	37.5	1.23	1.08	1.41	0.002
Less poor^b^	338	1158	29.2	Ref			
Age group (years)							
< 5	395	1177	33.6	1.76	1.46	2.14	< 0.001
5–14	227	427	53.2	2.74	2.27	3.31	< 0.001
≥ 15	117	624	18.8	Ref			
Sub-county							
Gem	278	278	278	1.38	1.14	1.65	0.001
Siaya	278	842	31.8	1.12	0.93	1.35	0.241
Rarieda	193	701	27.5	Ref			
ITN usage							
Yes	429	1449	29.6	0.84	0.73	0.96	0.010
No	29.6	779	39.8	Ref			

*ITN* insecticide-treated net, *SES* socioeconomic status

^a^‘Poorest’ was constituted by collapsing the poorest three quintiles

^b^‘Less-poor’ was constituted by collapsing the wealthiest two quintiles

**Table 4:** Association of socioeconomic status with care-seeking and medication usage in Siaya County, Kenya, 2012

	Care seeking (n = 1182)^a^							Medication use (n = 1180)^a^						
	n	N	%	aPR	95% CI		p value	n	N	%	aPR	95% CI		p value
SES														
Poorest	411	598	68.7	0.95	0.87	1.04	0.229	447	598	74.8	0.94	0.87	1.00	0.087
Less-poor	414	584	70.9	Ref				457	582	78.5	Ref			
Age group (years)														
< 5	535	716	74.7	1.27	1.14	1.40	< 0.001	582	714	81.5	1.27	1.15	1.40	< 0.001
5–14	124	188	66.0	1.11	0.97	1.29	0.120	142	188	75.5	1.17	1.04	1.32	0.008
≥ 15	166	278	59.7	Ref				180	278	64.8	Ref			
Sub-county														
Gem	301	407	74.0	1.05	0.94	1.17	0.391	326	406	80.3	1.10	1.01	1.20	0.037
Siaya	301	459	66.8	0.91	0.82	1.02	0.110	347	458	75.8	1.02	0.94	1.10	0.657
Rarieda	223	316	70.6	Ref				231	216	73.1	Ref			
Sex														
Female	460	656	70.1	1.03	0.96	1.11	0.350	500	655	76.3	1.02	0.95	1.08	0.617
Male	365	526	69.4	Ref				404	525	77.0	Ref			

*SES* socioeconomic status, *aPR* adjusted prevalence ratio, *CI* confidence interval

^a^Among the surveyed population who self-reported fever in the prior 2 weeks

Ownership of at least one ITN per household overall was very high at 93.9%; ITN ownership was not different between the poorest and less-poor households (93.5% versus 94.3, p = 0.72) (Table 5). The use of ITNs was also common; overall, 65.0% of persons reported using nets the night before the survey. In multivariate analysis of association between SES and ITN usage, 63.2% of persons in the poorest group used ITNs compared with 66.8% amongst the less-poor, but the difference was not statistically significant (aPR = 0.96; 95% CI = 0.90–1.02, p = 0.18). Significant differences were observed in reported ITN use by sub-county, with a significantly higher proportion of persons from Rarieda (80.5%) using ITNs compared to Gem (56.5%) or Siaya (59.1%) (p < 0.001 for both).

**Table 5:** Association between household socioeconomic status and insecticide-treated net use in Siaya County, Kenya, 2012

	n	N	ITN use percent	aPR	95% confidenceinterval		p-value
SES							
Poorest	676	1070	63.2	0.96	0.87	1.05	0.397
Less-poor	773	1158	66.8	Ref			
Age group (years)							
< 5	790	1117	67.1	1.00	0.94	1.07	0.976
5-14	231	427	54.1	0.79	0.71	0.89	< 0.001
≥ 15	428	624	68.6	Ref			
Sub-county							
Gem	387	685	56.5	0.70	0.63	0.79	< 0.001
Siaya	498	842	59.1	0.73	0.65	0.81	< 0.001
Rarieda	564	701	80.5	Ref			
Sex							
Female	802	1225	65.5	1.00	0.95	1.07	0.811
Male	647	1003	64.5	Ref			

*SES* socioeconomic status, *ITN* insecticide-treated net, *aPR* adjusted prevalence ratio

Of the 1180 individuals with reported history of fever in the 14 days prior to the survey who had malaria infection and had SES data available, 34.5% (n = 505) took the recommended first-line malaria medication, artemether-lumefantrine (AL) (Table 6). Among those who took AL, 30.9% were from the poorest households compared to 36.2% from less-poor households (p = 0.43). Amongst individuals who used any malaria medicines, use of non-recommended medicines was 4.9% in the poorest households compared to 3.5% in less-poor households (p = 0.32). The expenditure on any type of malaria medications in the 14 days prior to survey was not statistically different between the poorest and less-poor household members (mean US$0.35, standard deviation [30] US$0.52 versus mean US$0.40 [US$0.55]; p = 0.076, respectively). However, persons in the poorest households spent significantly more purchasing non-recommended malaria medicines compared to persons from less-poor households (mean = US$1.36 [US$0.91] versus mean US$0.98 [US$0.80]; p = 0.039).

**Table 6:** Use of and expenditure on malaria medication in surveyed population who reported fever in prior 2 weeks in Siaya County, Kenya, 2012

Utilization of medication (N = 1180)	All	Poorest households	Less-poor households	
	n (%)	n (%)	n (%)	p-value^c^
Artemether-lumefantrine	396 (33.6)	185 (30.9)	211 (36.2)	0.434
Sulphadoxine-pyrimethamine	8 (0.7)	6 (1.0)	2 (0.3)	0.119
Amodiaquine	11 (0.9)	7 (1.2)	4 (0.7)	0.284
Quinine	14 (1.2)	6 (1.0)	8 (1.4)	0.721
Chloroquine	5 (0.4)	3 (0.3)	2 (0.3)	0.557
Overall (any malaria medicine)	429 (36.4)	205 (34.3)	224 (38.4)	0.133
Non-recommended medicine^a^	38 (4.2)	22 (4.9)	16 (3.5)	0.332

	Mean (SD) in USD	Mean (SD) in USD	Mean (SD) in USD	
Expenditure on all malaria medications per person	0.38 (0.50)	0.35 (0.52)	0. 40 (0.55)	0.076

	Mean (SD) Median (IQR) in USD	Mean (SD) Median (IQR) in USD	Mean (SD) Median (IQR) in USD	p-value^d^
Expenditure on all medications per person among only those who paid for drugs (n = 424)^b^	1.04 (0.32) 1.01 (1.01–1.01)	1.02 (0.32) 1.01 (1.01–1.01)	1.05 (0.33) 1.01 (1.01–1.01)	0.345 0.926
Expenditure on non-recommended malaria medicines per person (n = 38)	1.14 (0.86) 0.62 (0.42–2.24)	1.36 (0.91) 1.43 (0.45–2.24)	0.98 (0.80) 0.62 (0.42–2.24)	0.039 0.018

*USD* United States dollars, *SD* standard deviation, *IQR* interquartile range

^a^Non-recommended medicine for malaria treatment included sulfadoxine-pyrimethamine, amodiaquine, quinine used by non-pregnant women and chloroquine

^b^Mean prices of adult formulation were artemether-lumefantrine = USD 1.01; sulfadoxine-pyrimethamine = USD 0.62; amodiaquine = USD 0.42; quinine = USD 2.24; chloroquine = USD 0.40

^c^Fisher’s exact test

^d^Wilcoxon rank sum test to compare medians and t-test to compare means; excludes children who received medicine for free from public health facilities

## Discussion

The study evaluated the relationship between burden of malaria infection and household SES within an area of rural western Kenya. This is the first published paper to assess the relationship between malaria indicators and SES using the MCA model to generate household wealth quintiles based on continuous and categorical variables. The findings show that individuals in the poorest households had a higher burden of malaria infection compared to those from less-poor households. Persons from the poorest households also spent significantly more money to purchase medications that are not recommended for malaria treatment, which are likely to have less clinical efficacy and lead to unnecessary risk of adverse effects and complications of taking inappropriate medications. No significant associations between care seeking and SES or medication use and SES were observed, and the study found high access to and use of ITNs, irrespective of household SES. Prevalence of malaria infection was significantly higher in Gem sub-county compared to Rarieda sub-county. This could be due to high vegetation coverage and the presence of River Yala which cuts across the sub-county. These findings contribute to the scarce published literature on malaria and socioeconomic inequalities. Although there is extensive literature on health inequalities and health outcomes more generally, no previous study has evaluated the relationships between malaria indicators and SES using MCA to analyse microeconomic data.

The study results are similar to findings by Somi and colleagues who reported a large variation in parasitaemia rates between socioeconomic groups, where individuals with the lowest SES were significantly more likely to have malaria parasites than less-poor individuals [8]. Findings from this analysis, however, contrast with those of de Castro and Fisher who found that SES had no association with malaria infection [6]. The de Castro study, however, was limited to children aged 6–59 months whereas a study by Somi et al. in which the analysis was not restricted to a specific age group [5, 8]. Both cross-sectional studies used household assets and proxies to measure SES using the PCA model [6, 10, 19]. This study addressed some of the limitations of these previous studies by using malaria confirmed by microscopy, as the main outcome of interest controlling for age group, and using generalized linear models instead of traditional concentration indices and Lorenz curves to estimate the risk of health indicators as a measure of inequity as recommended by World Bank and World Health Organization [13, 17, 19].

Previous studies on health outcomes, including malaria, and SES have traditionally used the PCA model to generate a household SES index. The PCA model relies heavily on dichotomous socioeconomic variables to achieve a composite household SES index [14, 15, 19]. Benefits of using the MCA model are inclusion of both continuous and categorical variables and larger weights for assets, which increases statistical power [21, 22]. Using PCA models to generate household SES indices was anticipated to facilitate a more robust evidence base for assessing the associations between health outcomes and poverty, especially at the household and community levels [14, 15, 19, 21]. However, recent literature has demonstrated the weaknesses of PCA models including the inability to accommodate continuous variables such as number of assets owned and generation of low asset weights, which makes it difficult to determine clear wealth quintile cut-offs particularly in settings where most households have the same assets and therefore the same or very similar SES scores [21, 22]. Based on evidence already published [20] that MCA is a better model than PCA, this study has applied MCA for assessment of socioeconomic status to assess malaria related health inequalities.

The study determined that nearly two-thirds of persons had fever in the past 2 weeks, and the majority (81%) of them took medication with an equal proportion of individuals among the poorest and less-poor households. Nearly half of those who sought care went to health facilities, but the other half sought care from pharmacies and informal drug shops. Research from Kenya shows that people who seek care from health facilities are more likely to get tested for malaria and receive the first-line recommended medications for treatment compared to those who go to pharmacies and informal drug shops [29, 31–33]. There were no differences observed in the expenditure on all malaria medications per person between SES groups, and less than 5% of persons purchased non-recommended malaria medicines overall, which is a positive finding. However, the poorest households spent more to purchase potentially ineffective medicines compared to less-poor households; ineffective treatments potentially prolong parasitaemia or fail to clear parasitaemia, which can lead to recrudescence or severe malaria and increased expenditures on additional treatments or hospitalizations. The findings of this study suggests a need to encourage healthcare seeking in the formal health sector, especially among the poorest households.

No significant differences in ITN ownership or usage between the poorest and less-poor households was observed in this rural western Kenya community in 2012, which was less than a year after the first universal coverage ITN distribution in Siaya County. Ownership of at least one ITN per household overall reached near full coverage (94%) and was well above the national target of 80% [34]. Subsequent national household surveys have consistently demonstrated significant differences in ITN ownership, access and use between the lowest and highest wealth quintiles [4, 26]. Because this cross-sectional study was conducted in a relatively small geographic area (i.e., three sub-counties) of rural Siaya County, there is probably much less socioeconomic variation compared to the national population and more uniformity in programmatic distribution within a single county.

A key principle of the Kenya Health Policy 2014–2030 is to achieve equity in the distribution of health services and interventions by 2030 [35]. Findings from this study illustrate existing socioeconomic inequalities in the burden of malaria infection and expenditures on non-recommended malaria medication in this rural western Kenya setting. However, the lack of differences between SES groups in care seeking, overall medication use and expenditures, and ITN ownership and use demonstrate the progress toward achieving equitable access to health services and distribution of free malaria commodities, including first-line medicines for treatment and ITNs, in western Kenya. Analysis of malaria indicators in relation to household SES using MCA methodology can be used to monitor progress towards achieving health equity goals in line with the Kenya Health Policy 2014–2030 and global sustainable development goals [36].

The study has a number of limitations. Findings were based on one cross-sectional survey preventing any evaluation of cause-and-effect of SES on malaria indicators over time. A longitudinal or trend analysis of repeated surveys would have provided an opportunity to study changes in SES and monitor the gap in malaria indicators between the poorest and less-poor households over time as malaria control interventions, including free first-line malaria treatment at health facilities and ITNs, were implemented. The other limitation was inclusion of only households with children < 5 years of age based on protocol-specific objectives. While this study advances the knowledge related to the association between malaria, control interventions and microeconomics, under these limitations, it reduces generalizability. Additionally, expenditures were calculated per person rather than per household because not all persons in the household were interviewed or tested for malaria. Although all children < 5 years of age were surveyed, only a small proportion of persons ≥ 5 years of age were included in the survey sample. Finally, the use of assets as proxies for SES also has limitations including, most importantly, that the monetary value of assets was not collected, and hence the net worth of the household might be over- or under-estimated. Asset-based proxies, however, have been shown as a reasonable way to measure wealth status in the absence of household income or expenditure data, which is not commonly available in informal economies [30]. This study did not compare the current results with any other results which could have been analysed using other methods besides MCA because there is already evidence that MCA is better model than PCA. Such comparison would not be statistically different in assigning households into the quintiles [20].

## Conclusion

In rural western Kenya, individuals in the poorest households had a higher burden of malaria prevalence compared to those from less-poor households. However, no significant differences were observed in care seeking, overall medication use and expenditure, or ITN ownership and use between households based on SES. This study demonstrates that the MCA model can be a useful tool for assessing malaria-related health inequalities at the microeconomic level and to monitor progress towards achieving equitable access to health services and distribution of malaria interventions in line with national and global health and development goals.

## Abbreviations

AL: artemether-lumefantrine
aPR: adjusted prevalence ratio
CDC: Centers for Disease Control and Prevention
DALY: disability-adjusted life year
HDSS: health and demographic surveillance system
IRB: institutional review board
ITN: insecticide-treated net
KEMRI: Kenya Medical Research Institute
MCA: multiple correspondence analysis
PCA: principal correspondence analysis
RDT: rapid diagnostic test
SES: socioeconomic status
SSC: scientific steering committee
